# Supplementary material for: Salinity Impacts the Functional mcrA and dsrA Gene Abundances in Everglades Marshes
Source: Microorganisms. 2023 Apr 30;11(5):1180. doi: 10.3390/microorganisms11051180 (PMC10223451; doi:10.3390/microorganisms11051180)
Supplement: Supplementary file 1 [file microorganisms-11-01180-s001.zip › Supplemental Data.pdf]

Supplemental Data: Salinity Impacts the functional *mcrA* and *dsrA* gene abundances in Everglades marshes

Supplemental Table S1: *mcrA* gene samples from brackish (BW) and freshwater (FW) sites with corresponding year of collection: initial (Y0) or 2 years later (Y2). Samples with no salt treatment (Control) or treated with saltwater pulses (Salt) are indicated in the treatment column. The Chambers indicated as controls were sampled at Y0 and again at Y2 while the treatment chambers were sampled only at Y2. Total Observed OTUs (Operational Taxonomic Units) indicate the total number of reads identified as a taxonomic cluster. Observed Unique OTUs indicate the richness within the sample as explained by the various diversity indices, Chao1, Shannon, Simpson, and Inverse Simpson.

Group	Treatment	Chamber	Total Observed OTUs	Unique Observed OTUs	Chao1	Shannon	Simpson	Inverse Simpson
**BW_Y0**	Control	3B	2740	466	1054.09	3.88	0.91	10.63
**BW_Y0**	Control	8B	2943	321	321.00	4.67	0.95	20.67
**BW_Y2**	Control	5B	2744	567	938.13	4.79	0.95	21.91
**BW_Y2**	Control	3B	2918	593	1303.03	4.31	0.92	12.26
**BW_Y2**	Control	7B	2831	488	730.31	4.43	0.94	17.29
**BW_Y2**	Control	8B	2616	709	1241.05	5.47	0.99	72.84
**BW_Y2**	Control	1B	2741	571	995.23	4.46	0.94	16.82
**BW_Y2_Saline**	Salt	11B	2810	369	369.00	4.52	0.94	16.48
**BW_Y2_Saline**	Salt	12B	2747	447	920.61	3.82	0.90	9.84
**BW_Y2_Saline**	Salt	13B	2780	548	968.00	4.86	0.96	27.12
**BW_Y2_Saline**	Salt	16B	2766	637	1248.85	4.63	0.95	19.41
**FW_Y0**	Control	2F	2879	26	26.00	3.19	0.96	22.39
**FW_Y0**	Control	6F	2770	534	44194.00	5.02	0.98	42.28
**FW_Y0**	Control	9F	2791	296	296.00	5.02	0.98	56.51
**FW_Y0**	Control	8F	2909	65	65.00	4.09	0.98	53.40
**FW_Y2**	Control	2F	2639	644	1039.00	5.46	0.99	92.54
**FW_Y2**	Control	8F	2880	736	91687.00	5.61	0.99	78.04
**FW_Y2**	Control	9F	2784	463	742.43	4.46	0.96	22.26
**FW_Y2**	Control	10F	2784	171	171.00	4.68	0.98	46.02
**FW_Y2**	Control	3F	2795	821	1461.06	5.57	0.98	53.38

**FW_Y2**	Control	6F	2923	458	458.00	5.58	0.99	111.63
**FW_Y2_Saline**	Salt	12F	2813	308	308.00	4.97	0.98	44.41
**FW_Y2_Saline**	Salt	15F	2916	693	1254.16	5.30	0.98	47.17
**FW_Y2_Saline**	Salt	16F	2760	546	761.47	4.52	0.94	16.46
**FW_Y2_Saline**	Salt	13F	2707	723	1392.77	5.40	0.99	69.07
**FW_Y2_Saline**	Salt	14F	2872	767	1434.26	5.64	0.99	77.37

Supplemental Table S2: *dsrA* gene samples from brackish (BW) and freshwater (FW) sites with corresponding year of collection: initial (Y0) or 2 years later (Y2). Samples with no salt treatment (Control) or treated with saltwater pulses (Salt) are indicated in the treatment column. The Chambers indicated as controls were sampled at Y0 and again at Y2 while the treatment chambers were sampled only at Y2. Total Observed OTUs (Operational Taxonomic Units) indicate the total number of reads identified as a taxonomic cluster. Observed Unique OTUs indicate the richness within the sample as explained by the various diversity indices, Chao1, Shannon, Simpson, and Inverse Simpson.

Group	Treatment	Chamber	Total Observed OTUs	Observed Unique OTUs	Chao1	Shannon	Simpson	Inverse Simpson
**BW_Y0**	Control	3B	7734	2130	5402.49	5.95	0.98	52.90
**BW_Y0**	Control	5B	7591	1312	2521.48	5.15	0.95	18.43
**BW_Y0**	Control	8B	7462	1071	1071.00	5.80	0.99	88.41
**BW_Y0**	Control	1B	7530	651	651.00	5.38	0.98	41.88
**BW_Y0**	Control	7B	7610	1530	2927.29	5.58	0.98	40.11
**BW_Y2**	Control	9B	7651	1897	3312.70	5.86	0.98	48.64
**BW_Y2**	Control	5B	7372	1113	1113.00	6.28	0.99	170.20
**BW_Y2**	Control	3B	7461	682	682.00	5.87	0.99	121.60
**BW_Y2**	Control	7B	7828	2991	9887.94	6.80	1.00	208.82
**BW_Y2**	Control	8B	7687	2394	4847.61	6.59	0.99	130.49
**BW_Y2**	Control	1B	7394	1814	602970.00	6.32	0.99	99.53
**BW_Y2_Saline**	Salt	14B	7602	1144	1144.00	6.33	0.99	179.84
**BW_Y2_Saline**	Salt	11B	7379	1252	1252.00	6.19	0.99	110.37
**BW_Y2_Saline**	Salt	12B	7389	1449	2351.21	5.55	0.97	37.78
**BW_Y2_Saline**	Salt	13B	7654	1177	1177.00	6.27	0.99	120.91
**BW_Y2_Saline**	Salt	16B	7682	2856	12800.67	6.85	1.00	204.65

**FW_Y0**	Control	3F	7545	1721	3053.44	6.04	0.98	63.78
**FW_Y0**	Control	2F	7476	1488	2414.88	5.87	0.99	71.32
**FW_Y0**	Control	6F	7664	1738	2831.79	6.20	0.99	118.47
**FW_Y0**	Control	9F	7673	1328	1328.00	5.95	0.98	58.45
**FW_Y0**	Control	8F	7503	1578	3033.00	5.68	0.97	32.65
**FW_Y2**	Control	2F	7434	1079	1445.34	4.97	0.95	20.33
**FW_Y2**	Control	8F	7407	1151	1151.00	5.94	0.98	53.50
**FW_Y2**	Control	9F	7709	1939	4389.58	5.58	0.95	19.04
**FW_Y2**	Control	10F	7686	1806	4191.54	5.89	0.98	54.47
**FW_Y2**	Control	3F	7472	615	615.00	5.41	0.98	41.01
**FW_Y2**	Control	6F	7637	807	807.00	5.95	0.99	124.16
**FW_Y2_Saline**	Salt	12F	7415	1535	367475.00	6.09	0.99	94.87
**FW_Y2_Saline**	Salt	15F	7663	2161	3288.05	6.44	0.99	105.01
**FW_Y2_Saline**	Salt	16F	7526	1705	3209.98	5.82	0.97	37.30
**FW_Y2_Saline**	Salt	13F	7441	1576	4073.50	5.62	0.98	44.34
**FW_Y2_Saline**	Salt	14F	7453	1260	1260.00	6.07	0.98	58.25

Supplemental Table S3: Summary of soil physiochemical properties of brackish and freshwater sites collected from porewater, and top 10 cm of soil cores at year 2. Welch's T-Test using alpha = 0.05. Significant differences are bolded.

	**Brackish Samples**			**Freshwater Samples**		
	**Mean**		**Significance**	**Mean**		**Significance**
**Extracellular Enzyme Activity**	**Control**	**Treatment**	**p value =**	**Control**	**Treatment**	**p value =**
**Alkaline phosphatase**	0.41	6.99E-05	0.36	3.82E-03	0.01	0.34
**Acid phosphatase**	2.80	2.61	0.87	3.89	3.51	0.83
**Arylsulfatase**	2.07	2.16	0.88	3.26	3.35	0.93
**Beta-1-4-glucosidase**	1.68	1.87	0.67	5.91	3.46	0.11
**Beta-1-4-cellobiosidase**	0.23	0.24	0.75	2.66	0.62	0.28
**Leucine amino peptidase**	0.01	0.00	0.36	0.01	0.04	0.10

**Bulk soil**						
**Phosphorus**	522.28	442.37	0.19	500.04	569.71	0.16
**Nitrogen**	2.47	2.17	0.10	3.25	3.29	0.74
**Carbon**	36.66	30.70	0.34	35.18	25.16	< **0.05**
**Porewater Biogeochemistry**						
**Temperature**	29.10	29.26	0.70	27.49	27.84	0.46
**Conductivity**	19.32	26.23	< **0.05**	0.51	3.80	< **0.05**
**Salinity**	11.49	16.00	< **0.05**	0.24	2.03	< **0.05**
**Alkalinity**	597.17	322.02	< **0.05**	155.00	165.67	0.17
**Chlorine**	6259.02	8937.19	< **0.05**	56.79	1230.81	< **0.05**
**DOC**	135.37	89.04	< **0.05**	22.83	22.62	0.94
**pH**	7.56	7.51	0.29	7.30	7.60	< **0.05**
**NH_4^+**	5.37	2.47	< **0.05**	0.45	0.93	0.07
**SO_4^{2-}**	362.81	964.63	< **0.05**	0.10	150.03	< **0.05**
**TDN**	8.84	4.58	< **0.05**	1.09	1.57	< **0.05**
**SRP**	5.47	2.76	< **0.05**	0.04	0.04	0.72
**TDP**	8.90	3.95	< **0.05**	0.32	0.34	0.54
**HS^-**	3.37	1.45	< **0.05**	0	0.08	< **0.05**

Supplemental Table S4: Summary of soil physiochemical properties comparing brackish and freshwater sites collected from porewater, and top 10 cm of soil cores at year 2 timepoint of study. Welch's T-Test using alpha = 0.05. Significant differences are bolded.

	**Site Samples**		
	**Mean**		**Significance**
**Extracellular Enzyme Activity**	**BW**	**FW**	**p value =**
**Alkaline phosphatase**	0.21	0.01	0.35
**Acid phosphatase**	2.70	3.66	0.39
**Arylsulfatase**	2.12	3.32	0.07
**Beta-1-4-glucosidase**	1.78	4.44	< **0.05**
**Beta-1-4-cellobiosidase**	0.23	1.43	0.10
**Leucine amino peptidase**	4.37E-03	0.03	0.06

**Bulk soil**			
**Phosphorus**	482.32	541.84	0.12
**Nitrogen**	2.32	3.27	< **0.05**
**Carbon**	33.68	29.17	0.21
**Porewater Biogeochemistry**			
**Temperature**	29.18	27.70	< **0.05**
**Conductivity**	22.78	2.49	< **0.05**
**Salinity**	13.74	1.32	< **0.05**
**Alkalinity**	459.59	161.40	< **0.05**
**Chlorine**	7598.10	761.20	< **0.05**
**DOC**	112.20	22.70	< **0.05**
**pH**	7.54	7.48	0.44
**NH_4^+**	3.92	0.74	< **0.05**
**SO_4^{2-}**	663.72	90.06	< **0.05**
**TDN**	6.71	1.38	< **0.05**
**SRP**	4.11	0.04	< **0.05**
**TDP**	6.43	0.34	< **0.05**
**HS^-**	2.41	0.05	< **0.05**

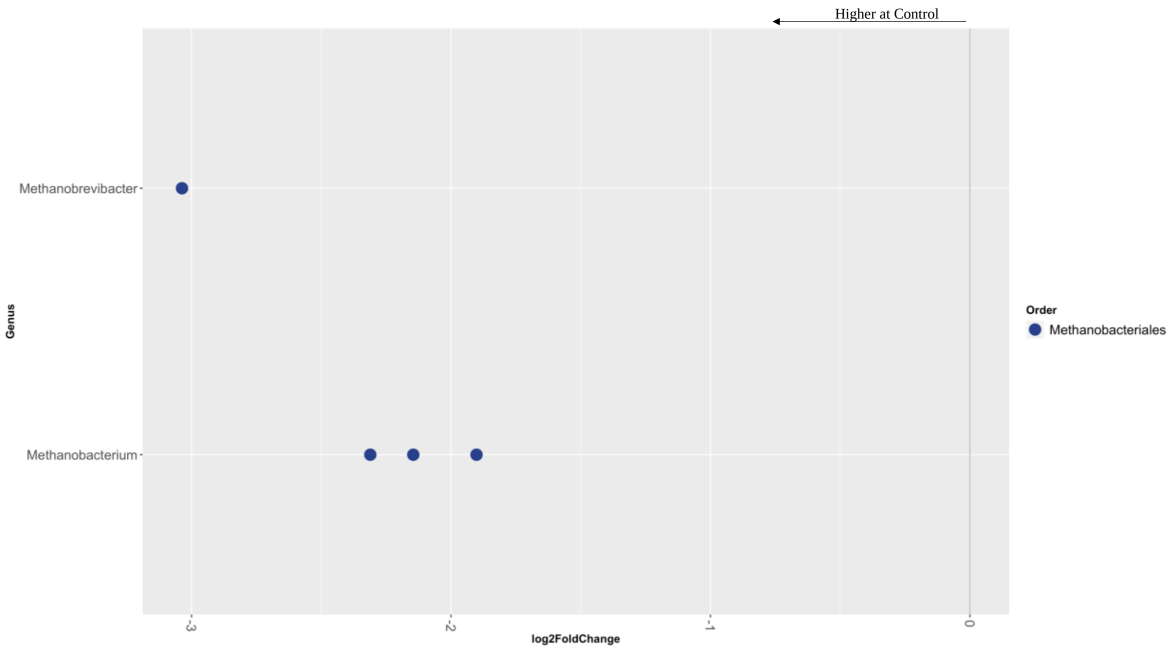

Supplemental Figure S1: Differentially abundant genera found at the Brackish site. Differential abundance plots between year 2 samples - control and saltwater treatment. Negative log fold changes represent significant differential abundance at the control samples. Decreases in abundance were observed in the Methanobacteriales Order after treatment.

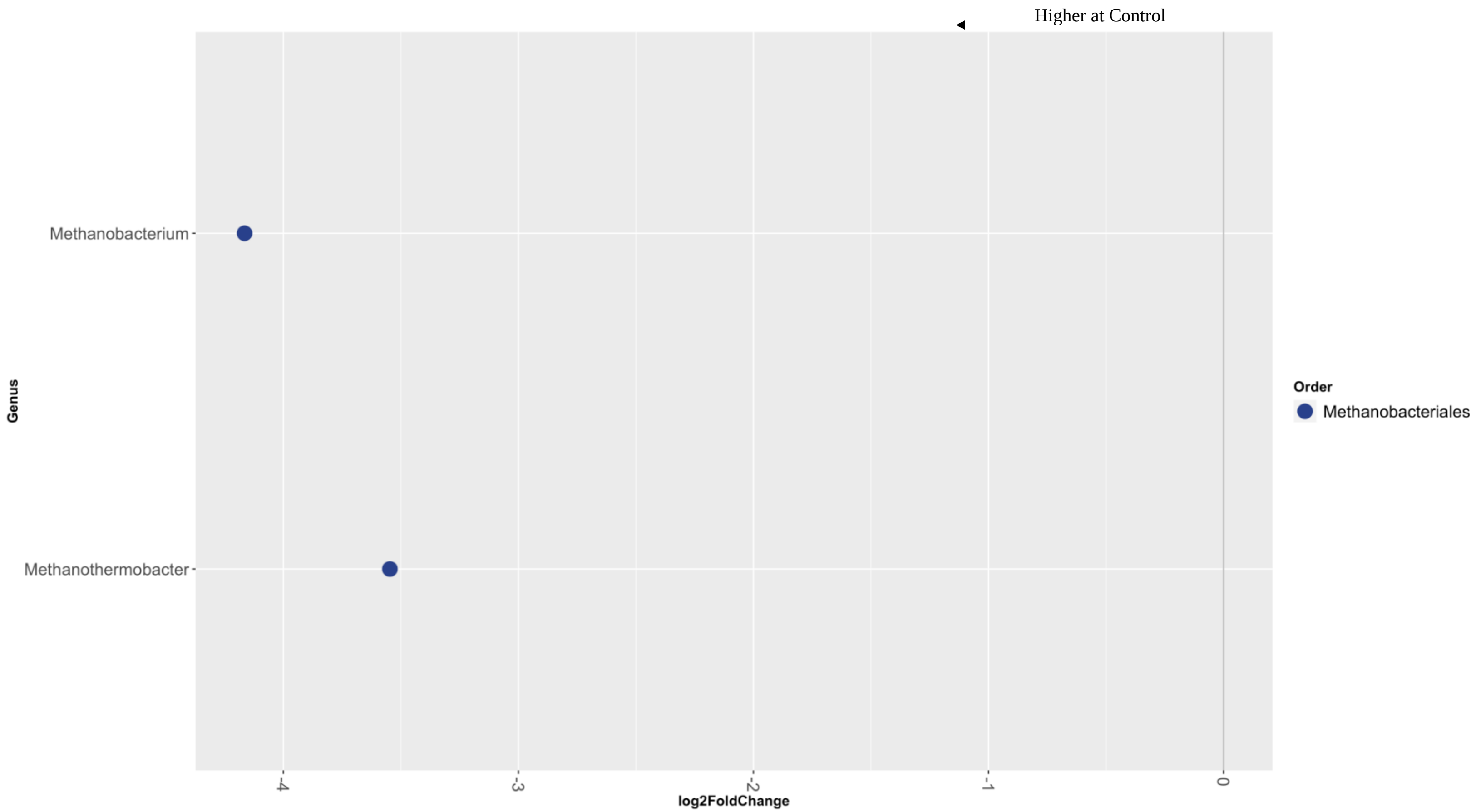

Supplemental Figure S2: Differentially abundant genera found at the Freshwater site. Differential abundance plots between year 2 samples - control and saltwater treatment. Negative log fold changes represent significant differential abundance at the control samples. Decreases in abundance were observed in the Methanobacteriales Order after treatment.
